# Physical and Microstructure Properties of Oyster Mushroom-Soy Protein Meat Analog via Single-Screw Extrusion

**DOI:** 10.3390/foods9081023

**Published:** 2020-07-31

**Authors:** Mazween Mohamad Mazlan, Rosnita A. Talib, Nyuk Ling Chin, Radhiah Shukri, Farah Saleena Taip, Mohd Zuhair Mohd Nor, Norazlin Abdullah

**Affiliations:** 1 Department of Process and Food Engineering, Faculty of Engineering, Universiti Putra Malaysia, Serdang 43400, Selangor, Malaysia; mazween.5624@gmail.com (M.M.M.); chinnl@upm.edu.my (N.L.C.); farahsaleena@upm.edu.my (F.S.T.); zuhair@upm.edu.my (M.Z.M.N.); 2 Department of Food Technology, Faculty of Science and Food Technology, Universiti Putra Malaysia, Serdang 43400, Selangor, Malaysia; radhiah@upm.edu.my; 3 Department of Technology and Natural Resources, Faculty of Applied Sciences and Technology, Universiti Tun Hussein Onn Malaysia, UTHM Pagoh Campus, Pagoh Higher Education Hub, KM 1, Jalan Panchor, Muar 84600, Johor, Malaysia; norazlinh@uthm.edu.my

**Keywords:** soy proteins, oyster mushroom, single-screw extrusion, apparent density, texturization index

## Abstract

Single-screw extrusion of a fibrous-structured meat analog from soy proteins added with low-grade oyster mushroom was successful. Satisfactory extrudates were obtained at a barrel temperature of 140 °C, screw speed range of 100–160 rpm, and oyster mushroom addition at 0%, 7.5%, and 15% via factorial experiments. Single-screw extrusion equipped with a slit die successfully produced expanded oyster mushroom-soy protein extrudates. However, the increase in the oyster mushroom content significantly decreased (*p* ≤ 0.05) the expansion ratio of the extrudate from 1.26 to 0.98. This result indicated that adding more oyster mushroom restrained the expansion ratio. The extrudates had a medium density range (max of 1393.70 ± 6.30 kg/m^3^). By adding oyster mushroom, the extrudates attained a higher moisture content (range = 34.77% to 37.93%) compared with the extrudates containing the protein mixture only (range = 26.99% to 32.33%). The increase in screw speed and oyster mushroom significantly increased (*p* ≤ 0.05) the water absorption index. The increase in the texturization index was significantly influenced (*p* ≤ 0.05) by oyster mushroom addition rather than the screw speed. A defined fibrous structure supported the high texturization index and small shape of air cells observed in the extrudates.

## 1. Introduction

The combined effects of an increasing human population worldwide, awareness on animal protein sustainability, environmental concerns, and human health considerations are expected to increase the global market venue of plant protein-based meat to USD 7.5bn by 2025 [1]. Moreover, society has become interested in replacing animal protein sources with plant protein. This change in trend is due to the health benefits of plant protein-based meat analogs, which are low in saturated fats and sodium, cholesterol-free, and an excellent source of protein that is comparable to animal meat [2,3]. Recent studies have developed a nutritious plant protein-based meat analog that mimics the aesthetic quality (structure, taste, and appearance) of animal meat. These studies have attempted to develop plant protein-based meat analogs from different sources of protein (e.g., soybean, peanut, oilseed, cereal, and mycoprotein) [2,4]. Different cooking techniques (e.g., single-screw extrusion, twin-screw extrusion, Couette cell technology, and electrospinning) were applied [5,6,7,8]. However, substantial challenges remain in operation to achieve the right texture, appearance, and nutrient content of meat analogs. Among the main challenges are determining suitable processing parameters and feed compositions in the production of meat analogs. 

Extrusion is a continuous process of mixing, kneading, and shaping that involves both thermal and mechanical energies. It is a versatile cooking technique that can be applied to produce varieties of food such as cereals, meat analogs, and ready-to-eat food. Extrusion is a high-temperature/short-time process that is remarkably adaptable in fulfilling consumer demand for attractive products. The production of various food products is workable by simultaneously adjusting the conditions of feed ingredients and the setting parameters of the extruder. Feed ingredients generally consist of multiple components that will experience different structural transformations under extrusion cooking in the presence of heat, shear force, and water. In the extrusion of proteins, their original states are disrupted (denaturation of protein) and altered (realign and crosslinking) by high temperature, shear, and pressure. However, protein texturization is also dependent on the type of ingredient used, as the ingredients can enhance or inhibit the desired final product quality. Several authors have considered the effects of adding extra ingredients (e.g., flavor enhancer, starch, fiber, and microalgae) on the final quality (e.g., expansion, density, color, texture, and water absorption capacity) of meat analogs [5,6,9,10]. These studies have reported that the presence of additional ingredients in protein-based mixtures can positively and negatively affect the physical quality and improve the nutrient content of meat analogs. 

Mushrooms have been used as meat replacers in human diets due to their good source of macronutrients (protein and fiber) and micronutrients (essential amino acids, vitamins, and essential minerals), as well as low fat, sodium, and energy contents [2,11,12,13]. Mushrooms have been substituted in the development of beef/chicken patties and nuggets to develop healthier protein foods with a good appearance, taste, and texture [14,15,16]. The extent to which mushrooms can be added into plant-based meat analogs remains unclear. A previous study conducted by Ahirwar et al. [17] reported the effect of fresh button mushroom addition in a wheat gluten-based meat analog through the steaming method. However, their study was only limited to the hardness and nutritional properties of the meat analog. 

Low-grade oyster mushroom can be classified into the no market value category by the Federal Agricultural Marketing Authority, Malaysia [18]. They are small in cap size (ranging from 3 to 5 cm) and deformed in shape. Thus, they are usually sold at a much lower price (reduced by up to 60% per kilogram) than higher grades or are discarded as mushroom waste. Despite their physical flaws, low-grade oyster mushroom still has the same nutritional values (high protein and fiber) as its high-grade counterpart. Adding these low-grade mushrooms into meat analogs can help in producing value-added products. The combined effect of low-grade oyster mushroom addition and the extrusion setting parameters on the properties of a soy protein-based meat analog were assessed in this study via single-screw extrusion. In this study, the oyster mushroom-soy protein meat analog was unpuffed, contained medium moisture (30% < moisture content < 50%), and presented a fibrous structure. Therefore, the set objectives were to (i) determine a range of suitable extrusion process parameters that satisfy the extrusion performance and extrudates’ specific criteria and (ii) evaluate the effects of different screw speeds and oyster mushroom addition on the physical properties (expansion ratio, apparent density, moisture content, water absorption index (WAI), and texturization index (TI)) and microstructure of the extrudates.

## 2. Materials and Methods

### 2.1. Materials

Low-grade gray oyster mushrooms (*Pleurotus sajor-caju*) were obtained from Ladang Tanaman Cendawan, Universiti Putra Malaysia, Serdang. Soy protein concentrate (SP) and isolated soy protein (ISP) were purchased from Shaanxi Jintai Biological Engineering Co., Ltd., China, and Imaherb Biotech Co., Ltd., China, respectively. Commercial wheat flour produced by Syarikat Faiza Sdn. Bhd., regular single refined rock salt manufactured by Halagel (M) Sdn. Bhd., and cooking palm oil produced by FFM Berhad were purchased from a local shop in Serdang, Malaysia. Additives such as food-grade soy lecithin and sodium metabisulfite were acquired from Evergreen and Engineering Resources, Malaysia. These additives were used to improve the texture of extrudates. Distilled water was obtained from the Packaging and Preservation Laboratory, Universiti Putra Malaysia, Malaysia.

### 2.2. Processing of Gray Oyster Mushroom

The fresh low-grade oyster mushrooms were washed to remove dirt and unwanted particles, and they were left in a strainer for 15 min to remove excess water. They were then chopped into mashed mushrooms using an electrical chopper (HR1393/01, Philip, Malaysia) for 1 min. On the basis of the analyses performed according to the Official Methods of AOAC 16th Edition (1995) [19], the low-grade oyster mushroom contained 91.6 moisture, 3.3 protein, <0.1 fat, 4.5 carbohydrate, 0.6 ash, and 3.0 g/100 g dietary fiber. 

### 2.3. Preparation of Feed Mixtures

A base mixture was prepared by mixing 50% of SP and 50% ISP. Additional ingredients such as wheat flour, soy lecithin, sodium metabisulfite, salt, cooking palm oil, distilled water, and oyster mushroom were added on a weight percentage basis of the base mixture. Five feed mixtures were prepared at different percentages of oyster mushroom content: 0% (acts as control), 7.5%, 15%, 25%, and 35%. The formulation ingredient details of the five feed mixtures on a weight basis (wb%) are given in Table 1. These feed mixtures were stirred by using a kitchen hand mixer and kept in airtight containers. The feed mixtures were refrigerated overnight before performing extrusion to achieve uniform hydration. 

### 2.4. Extrusion Cooking 

Extrusion cooking was carried out using a laboratory-scale single-screw Brabender 19/20D extruder (Brabender GmbH and Co., Duisburg, Germany). The extruder has a grooved barrel with a length–diameter ratio of 20:1, two heating zones, and a heating element at the extruder’s die head. A slit extrusion die with a dimension of 20 × 2 mm (width × height) was attached to the extruder discharge. Different extruder temperatures were set at 80 °C (feeding zone), variable temperature (130−160 °C; compression zone), and 45 °C (die) for the first zone, second zone, and at the die, respectively. A compression screw (compression ratio 2:1) was used to extrude the meat analog. Feed mixtures were fed through a standard stainless steel hopper with a dosing screw rotating at a constant speed of 25 rpm. The extrusion screw speed was set between 110 and 160 rpm. The extrudates discharged at the die were only collected once extrusion reached a steady state as indicated by the consistent values of an extruder’s torque and die temperature (Zone 3) recorded in the extrusion torque–temperature over time profiles. Extrudate samples were left to cool down to room temperature before being packed in polyethylene Ziplock bags. The extrudates were kept in a freezer at −18 °C until further analysis.

### 2.5. Factorial Experiment Design

A range of suitable extrusion process parameters was selected based on satisfactory evaluation, which meets extrusion and the product’s specific criteria. The evaluation was categorized into two criteria: (1) the observation of the extrusion ability and (2) extrudate’s physical appearances (e.g., surface texture and fibrous structure), for each case of extrusion conditions. Thus, the factorial experimental design was applied to conduct a preliminary investigation for this selection purpose. Three extrusion process variables, namely barrel temperature, screw speed, and oyster mushroom percentage, were considered. The first stage was to determine the suitable extruder barrel temperature (compression zone). Feed mixture containing 0% oyster mushroom (control) was extruded at four levels of barrel temperature (130, 140, 150, and 160 °C) and six levels of screw speed (110, 120, 130, 140, 150, and 160 rpm). The extruder barrel temperature that exhibited a smooth extrusion flow produced unpuffed extrudates with a smooth surface and fibrous structure. These extrudates were then further applied to the second stage of the preliminary investigation to determine the suitable percentage of oyster mushroom addition. Feed mixtures containing 7.5%, 15%, 25%, and 35% oyster mushroom were extruded at the selected extruder barrel temperature and at the same six levels of screw speed. The obtained extrusion flow behavior and physical appearances of each extrudate were summarized using Microsoft Excel 2016 (Vista Edition, Microsoft Corporation, Albuquerque, NM, USA).

### 2.6. Characterization of Oyster Mushroom-Soy Protein Extrudate Physical Properties

#### 2.6.1. Expansion Ratio 

Expansion ratio was calculated using Equation (1), in which the dimension of the extrudate (width: mm × height: mm) was divided by the flat sheet die dimension (width × height: 20 × 2 mm). A digital Vernier caliper (Mitutoyo Corp., Kawasaki, Japan) was used to measure three replicates of the sample dimension. The average expansion ratio measurement was recorded.

(1) $$\mathrm{Expansion}\;\mathrm{ratio}\;(\mathrm{ER})=\frac{{\mathrm{Extruded}\;\mathrm{sample}\;\mathrm{dimension}\;(\mathrm{mm}}^{2})}{{\mathrm{Flat}\;\mathrm{sheet}\;\mathrm{die}\;\mathrm{dimension}\;(\mathrm{mm}}^{2})}$$

#### 2.6.2. Apparent Density

Equation (2) was applied to calculate the apparent density of the extrudate. The width, height, and length of the extrudate were measured by using a digital Vernier caliper (Mitutoyo Corp., Kawasaki, Japan). An analytical balance (Mettler Toledo, Greifensee, Switzerland) was employed to measure the extrudate’s weight. The apparent density of three replicates was obtained as an average.

(2) $${\mathrm{Apparent}\;\mathrm{density}\;(\rho }_{\mathrm{app}})=\frac{4W}{{\pi D}^{2}L}$$

where 

W = weight of extrudate (kg); 

$D^{2}$ = dimension of (width × height) ($m^{2})$; 

L = length of the extrudate (m);

$\rho_{\mathrm{app}}$ = apparent density of the extrudate ($\mathrm{kg}/m^{3})$.

#### 2.6.3. Water Absorption Index 

The WAI was determined following the procedure of Anderson [20]. A suspension was prepared by mixing 2.5 g of ground sample (No. 18 mesh) with 30 mL of distilled water in a 50 mL centrifuge tube and gently stirring for 30 min at room temperature. The suspension was centrifuged in a Universal 320 Hettich centrifuge (Andreas Hettich GmbH and Co., Tuttlingen, Germany) at 3000 rpm for 15 min [21]. The weight of sediment left in the centrifuge tube was recorded, and the WAI was calculated using Equation (3). The WAI was determined in triplicate for each sample, and the average was reported.

(3) $$\mathrm{Water}\;\mathrm{absorption}\;\mathrm{index}\;(\mathrm{WAI})=\frac{\mathrm{Weight}\;\mathrm{of}\;\mathrm{sediment}\;\mathrm{formed}\;(g)}{\mathrm{Weight}\;\mathrm{of}\;\mathrm{the}\;\mathrm{sample}\;(g)}$$

#### 2.6.4. Moisture Content

The moisture content of the extrudate was determined immediately after the extrudate exits the extruder’s die. The MX-50 Moisture Analyzer (Mettler Toledo, Greifensee, Switzerland) was employed to measure the moisture content. Before measurement, 2 g of the extrudate was heated at 105 °C until a constant weight was achieved. The mean of triplicate moisture content measurements was recorded.

#### 2.6.5. Texturization Index

**Figure 1:**
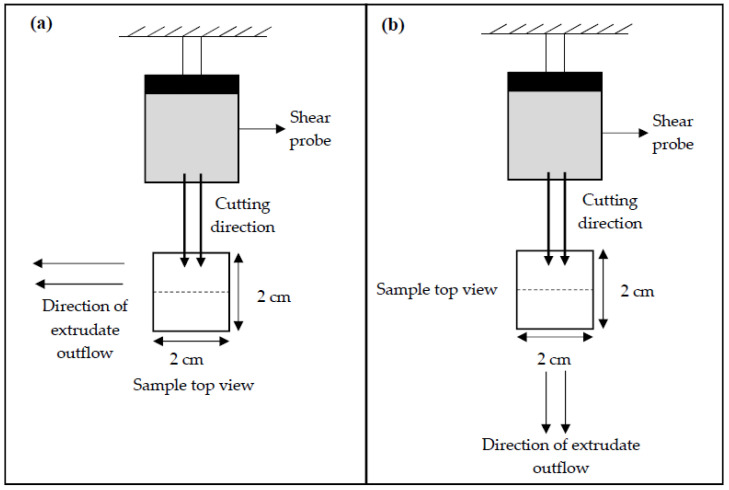
Test setup for the determination of (**a**) crosswise strength $F_{V}$ and (**b**) lengthwise strength $F_{L}$

The TI was determined following the procedure reported by Wu et al. [22], with some modifications in the test setting parameters of TA.XT Plus Texture Analyzer (Stable Micro Systems, Surrey, UK). For sample preparation, the samples (e.g., extrudate, hydrated textured vegetable protein (TVP), and chicken meat) were cut into squares (2 × 2 cm). They were cooked in water at 100 °C for 30 min and left to cool down at a surrounding temperature of 28 °C ± 1 °C for 30 min. Each sample was sheared using a Warner–Bratzler stainless steel shear probe (shear angle 60°, probe thickness: 1.00 mm) to 100% of its original thickness at a speed of ${1\mathrm{mms}}^{-1}$ along the vertical (lengthwise strength, $F_{L})$ and parallel directions (crosswise strength, $F_{V})$ to the direction of extrudate outflow from the extruder [22,23]. The ratio of lengthwise strength $F_{L}$ and crosswise strength $F_{V}$ expressed the TI (Equation (4)). TI measurements were repeated three times for each cutting direction, and the data were averaged. Figure 1 illustrates the cutting directions for the lengthwise strength and crosswise strength measurement.

(4) $$\mathrm{Texturization}\;\mathrm{index}\;(\mathrm{TI})=\frac{{\mathrm{Lengthwise}\;\mathrm{strength},\;F}_{L}\;\left(g\right)}{{\mathrm{Crosswise}\;\mathrm{strength},\;F}_{V}\;\left(g\right)}$$

#### 2.6.6. Scanning Electron Microscopy Analysis.

Scanning electron microscopy (SEM) analysis was conducted to observe fibrous structures and air cell formations in the extrudates. Extrudates were cut into the dimensions of 1 × 1 × 0.2 cm. For each test extrudate, they were cut in transversal and longitudinal directions for further observations. The test samples were mounted onto aluminum stubs by using double-sided carbon tape. Both sample surfaces of cutting directions were viewed under low-vacuum mode in a JSM-IT100 InTouchScope™ Scanning Electron Microscope (JEOL, USA) at 40× and 100× magnification with an accelerating voltage of 3 kV for raw extrudates. The low accelerating voltage was applied to minimize the charging effects on the test sample. 

#### 2.6.7. Data Analysis

A two-factor full factorial design was performed to determine the influences of the extrusion process parameter on the extrudate’s physical properties by using Minitab 17 software (Minitab Inc., State College, PA, USA). One-way analysis of variance was carried out to determine the level of significance at *p* ≤ 0.05. The Pearson coefficients (r) of linear correlation among the physical properties of extrudates were also determined. Tukey’s multiple comparison test was used to determine the significant difference at *p* ≤ 0.05 among a set of mean.

## 3. Results and Discussions

### 3.1. Evaluation of Extrusion Ability and Physical Characteristics of Oyster Mushroom-Soy Protein Extrudate 

**Figure 2:**
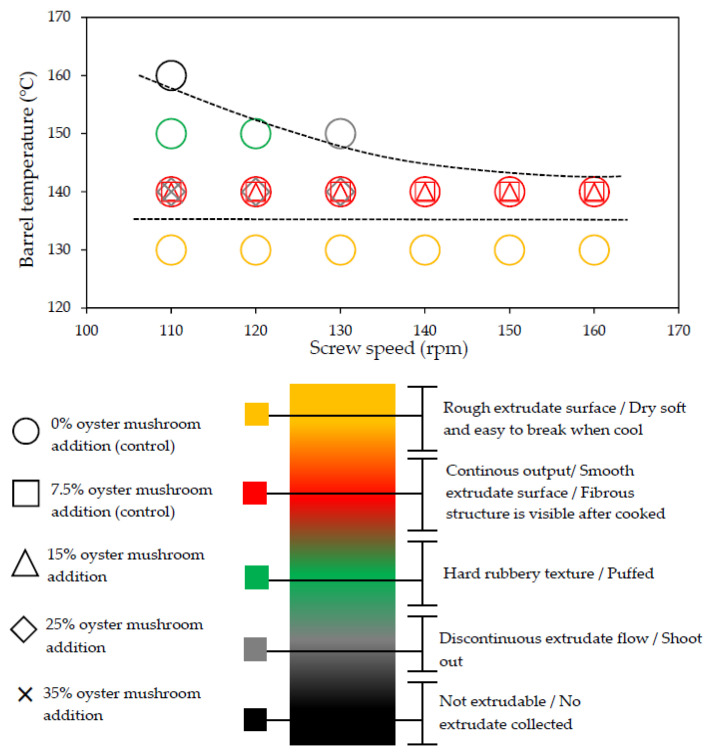
Summary of ranges of extrusion process conditions that categorize key extrusion selection criteria, namely extrusion ability and extrudate physical characteristics, as a function of barrel temperature and screw speed at five different levels of oyster mushroom percentages.

Extrusion parameters and performance can vary depending on the ingredients used and target extrudate characteristics. Therefore, a satisfactory evaluation can be conducted in preliminary investigations to identify the extrusion process variables (temperature, screw speed, and ingredient formulation) [5]. Table 2 summarizes the satisfactory evaluation results for the selection of suitable extrusion parameters to produce continuous unpuffed extrudates with a fibrous structure. A range of extrusion parameters for oyster mushroom-soy protein meat analog production is graphically shown in Figure 2. The suitable extrusion parameters were determined based on the extrusion ability and satisfactory extrudate’s physical appearance produced at various barrel temperatures, screw speeds, and oyster mushroom addition. Thadavathi et al. reported that a continuous extrudate output is limited to moisture content saturation and critical extrusion temperature [5]. In the current study, protein denaturation occurred at its critical extrusion temperature; therefore, the control feed mixture (0% oyster mushroom) was extruded at different barrel temperatures (130, 140, 150, and 160 °C; Figure 2). Extrusion of the control feed mixture at the barrel temperature of 130 °C exhibited intermittent extrudate flow and soft extrudates. However, the control feed mixture extruded at 140 °C provided a continuous output with a smooth extrudate surface and presented fibrous structure formation. The extrudate puffed at the die when the barrel temperature was 150 °C but began to shoot out at a screw speed of 130 rpm, which exhibited a dissatisfactory extrusion ability characteristic such as inconsistent output. The control feed mixture was not extrudable at 160 °C. The dough hardened at the die exit, and no extrudate was collected (Figure 2). Thus, the extruder barrel temperature of 140 °C was selected for feed mixture extrusion with the addition of oyster mushroom from 7.5% to 35%. The continuous output of extrudates with a smooth surface was restricted to feed mixture containing 15% oyster mushroom. At both 25% and 35% oyster mushroom addition, the extrudate flowed out inconsistently and shot out from the die. These formulations also presented dissatisfactory extrudability. Therefore, the extrusion parameters of a barrel temperature of 140 °C, screw speed (110–160 rpm), and oyster mushroom addition (7.5% and 15%) were only selected for further experiments. The physical characteristics of oyster mushroom-soy protein extrudate produced from the chosen extrusion parameters were analyzed. The results of these physical analyses are presented in Table 3.

### 3.2. Oyster Mushroom-Soy Protein Extrudate’s Physical Properties under Selected Extrusion Process Conditions

#### 3.2.1. Expansion Ratio and Apparent Density

In this study, single-screw extrusion equipped with a slit die of feed mixture produced expanded oyster mushroom-soy protein extrudates. This finding was consistent with that of Rehrah and Samard, who obtained expanded meat analogs from the extrusion of a protein-based mixture employed with a slit die [4,24]. As shown in Table 3, the expansion ratio of the extrudate ranged from 0.98 to 1.26. The expansion ratio of the extrudate was in between the expansion ratio of a peanut-based meat analog (0.67–1.33) as reported by Rehrah et al. [4]. The extrudate with no oyster mushroom had the highest expansion ratio of 1.26. Meanwhile, the addition of 15% oyster mushroom in the extrudate had the lowest expansion ratio of 0.98. These results indicated that adding more oyster mushroom content restrained the expansion of the extrudates even though the samples were extruded at the same screw speed of 160 rpm. Increased screw speed significantly increased the expansion ratio of the extrudates (*p* ≤ 0.05; Table 4). However, there was no increase between the expansion ratios of extrudates added with 15% oyster mushroom (Table 3) for all screw speeds. According to Rehrah [4], a low-expansion extrudate is likely to have a strong bond between protein molecules. The expansion ratio increased with increasing screw speed due to the formation of a high number of air cells. At a high extrusion screw rotation, the thermal energy and barrel pressure increased due to the shearing of the dough. Therefore, a high level of superheated steam and pressure in the dough was also developed [25]. When the dough emerged from the die, moisture in the dough flash-off and extrudate expanded due to the rapid pressure drop from high die pressure to atmospheric pressure [26]. In Table 4, there was a significant reduction in the expansion ratio when oyster mushroom addition increased to 15% (*p* ≤ 0.05). This result showed that increased oyster mushroom retarded extrudate expansion, possibly because of the presence of high protein, fiber, and fat contents in the feed. In the present study, oyster mushroom was added into the feed mixture because of its rich source of dietary fiber and protein [11,13]. Recent studies reported that a decrease in extrudate expansion increases the fiber and protein contents in the development of textured vegetable protein [4,10].

No significant negative correlation was found between the expansion ratio and apparent density of the extrudates (Table 5). The extrudates’ apparent density significantly decreased (*p* ≤ 0.05) when the screw speed and oyster mushroom increased (Table 4). The data in Table 3 showed that the extrudate with no oyster mushroom was denser (1380.96 ± 10.12 kg/m^3^) than the extrudate produced with 15% oyster mushroom addition (1272.73 ± 2.94 kg/m^3^). The apparent density of the extrudates was more likely affected by the increment of screw speed from 100 to 160 rpm than the addition of oyster mushroom. The apparent density of the extrudate made from soy proteins and oyster mushrooms was lower than that of the extrudate added with flavor enhancer as reported by Milani et al. [9]. Nevertheless, the apparent density of the extrudate was within the acceptable range of products, where the textured vegetable protein should have medium density, as mentioned by Rehrah et al. [4].

#### 3.2.2. Moisture Content

Table 3 presents the moisture content of the extrudates after they exited the extruder’s die. The moisture content of the extrudates containing oyster mushrooms was significantly high (*p* ≤ 0.05). However, no remarkable changes in the moisture content of extrudates run at the screw speed of 110–150 rpm were observed. A reduction in moisture content was observed for the extrudate produced with 0%, 7.5%, and 15% oyster mushroom at the speed of 160 rpm. These trends showed that the moisture content was negatively affected by screw speed but positively affected by oyster mushroom addition at a significance level of *p* ≤ 0.05 (Table 4). A strong negative correlation was found (*p* ≤ 0.05) between moisture content and expansion ratio, but no significant positive correlation was observed between moisture content and apparent density (Table 5). The less expanded oyster mushroom-soy protein extrudate contained higher moisture content. The moisture content of extrudates ranged from 26.99% to 37.93%. These results were consistent with data obtained in the development of a peanut-based meat analog from single-screw extrusion [4]. However, the moisture content was higher compared with those measured by Omohimi et al. [21] and Samard et al. [24]. They obtained a low moisture content of the extrudate (≤20% moisture content) by applying additional treatments to the feed ingredients before extrusion or to the extrudate after extrusion. These treatments were needed for storage conditions (e.g., at room temperature) and prolonged the shelf life of extrudates. 

#### 3.2.3. Water Absorption Index 

WAI is defined as the measure of the maximum volume occupied by polymer granules after hydration over water [27,28]. This index also represents the juiciness of a meat analog, which is an essential texture quality [4,21]. Table 3 shows the WAI ranging from 2.91 to 4.04. The WAI of extrudates significantly increased (*p* ≤ 0.05) with the increase in screw speed and oyster mushroom addition (Table 4). The results of correlational analysis showed that WAI was negatively correlated (*p* ≤ 0.05) with the expansion ratio and apparent density but positively correlated (*p* ≤ 0.05) with the moisture content and TI of the extrudate (Table 5). The high WAI of the extrudate may be dependent on the protein source [27,29]. The increase in the addition of oyster mushroom is expected to increase the protein level, which allows more water absorption to occur. This observation was in agreement with the study completed by Rehrah et al. [4], who reported that increased protein content increases the WAI of peanut-based meat analogs. The effect of increased screw speed and WAI was observed on the extrudate without oyster mushroom addition. The most expanded and less dense extrudate produced at the highest screw speed (160 rpm) had the highest WAI (Table 3). Similar findings were reported by Lin et al. [27], who found that an expanded, less dense, and high-moisture soy protein meat analog had the highest water absorption capacity than its counterparts.

#### 3.2.4. Texturization Index

The fibrous structure formation in a meat analog can be expressed by the TI with a dimensionless value of >1. The TI is the ratio of the maximum cutting strength in the vertical (lengthwise strength, $F_{L})$ and parallel (crosswise strength, $F_{V})$ directions to the direction of extrudate outflow from the extruder [23,30]. The maximum cutting strength was obtained from the maximum peak of shear force of the probe to pass through the fibrous structure in the extrudate, as generated by a force–deformation curve. The TI significantly increased (*p* ≤ 0.05) with increasing percentage of oyster mushroom addition (Table 4). Extrudates containing 7.5% and 15% oyster mushroom had a higher TI compared with the control extrudates. Thus, the presence of oyster mushroom in the formulations increased the formation of fibrous structure in the extrudates (Table 1). 

**Figure 3:**
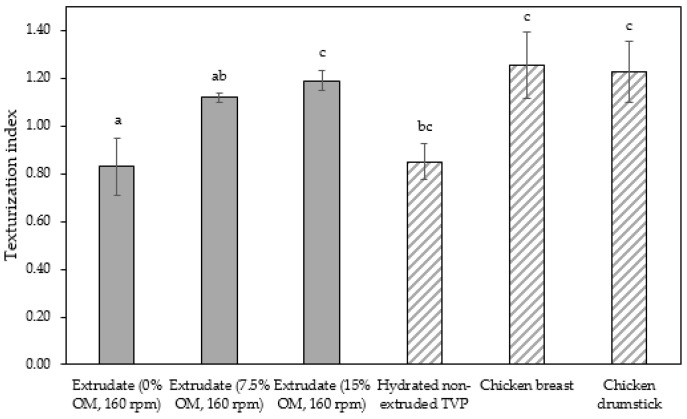
Texturization index of extrudates containing 0%, 7.5%, and 15% oyster mushroom compared with hydrated textured vegetable protein (TVP) and chicken meat (breast and drumstick). Note: Different superscript indicates significant difference at *p* ≤ 0.05.

The screw speed insignificantly affected the TI values of extrudates produced at speeds ranging from 110 to 160 rpm, but TI variation was observed. As shown in Table 3, the TI of the control extrudate showed an increase at the screw speed of 110–140 rpm and then dropped beyond this range. The TI of the extrudates containing 7.5% and 15% oyster mushroom decreased as the screw speed increased but then increased at the screw speed of 160 rpm. These findings suggested that protein polymerization in the dough occurred at the same denaturation temperature of 140 °C but differed with screw speeds. The TI was dependent on the extruder parameters (e.g., type of extruder, screw configuration, rotation speed, barrel temperatures, and feed rate) and feed conditions (e.g., ingredients and moisture content) applied. Numerous studies have investigated the effect of these independent variables on the TI of soy protein-based meat analogs [10,22,23,30,31,32]. The TI of the oyster mushroom-soy protein meat analogs ranged from 0.77 to 1.19 (Table 3). The TI of the extrudate achieved the same range of TI (0.7–1.2) as presented by Chen et al. and Fang et al. on meat analogs made from soy protein without additional ingredients, which were produced by a twin-screw extruder [23,31]. However, several studies have reported a higher range of TI (1.3–1.7) in soy protein-based meat analogs added with wheat gluten and extruded via twin-screw extrusion [22,30]. Figure 3 illustrates the TI of extrudates containing 0%, 7.5%, and 15% oyster mushroom as compared with the TI of hydrated TVP and chicken meat (breast and drumstick). The TI of the extrudates (0.83–1.19) was lower than that of chicken meat (~1.23–1.26) but higher than that of hydrated non-extruded TVP (~0.85). Comparison between extrudates with 15% oyster mushroom and chicken meats showed an insignificant difference in the TI value. Previous research has reported that chicken meat (e.g., chicken breast) has a higher TI due to large muscle fibers compared with extruded TVP [30,33]. 

#### 3.2.5. Microstructural Properties of Oyster Mushroom-Soy Protein Extrudate 

**Figure 4:**
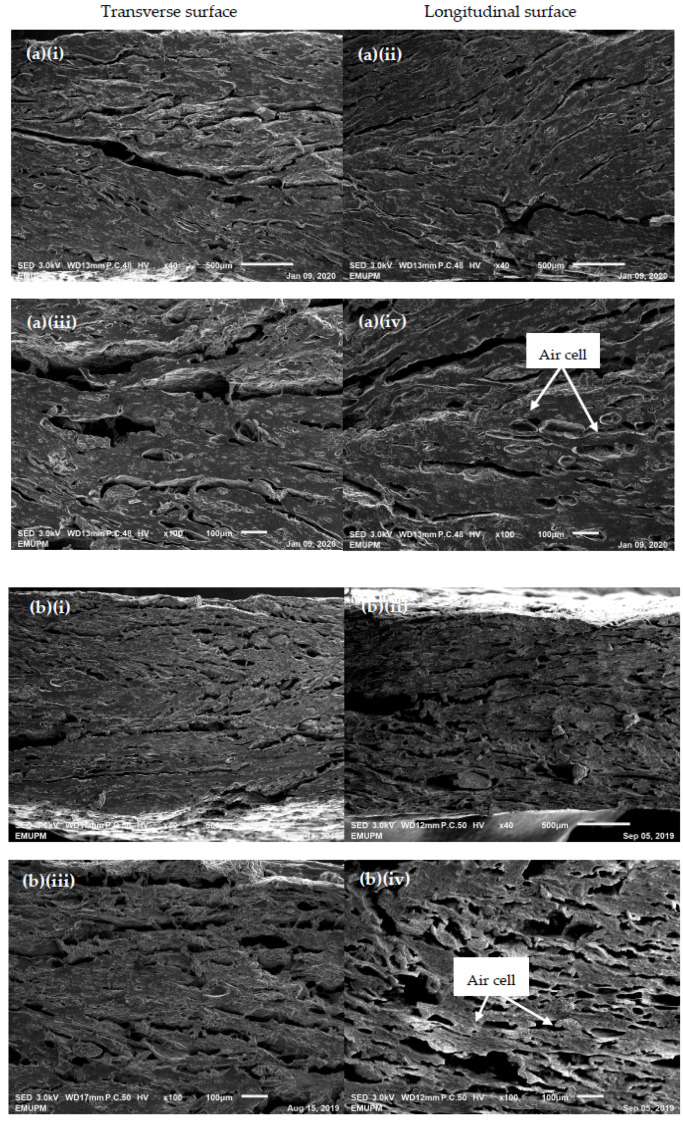
SEM images of raw extrudates without oyster mushroom (**a**: **i**–**iv**) and with 15% oyster mushroom (**b**: **i**–**iv**) produced at 160 rpm screw speed: transverse and longitudinal surfaces.

The oyster mushroom-soy protein meat analog microstructures in transverse and longitudinal surfaces are presented in Figure 4. The SEM images show that the extrudates produced from a short-slit die had a large number of air cells and non-uniform air cell distribution. This observation mirrored those of the previous studies that have investigated the microstructures of low-moisture meat analogs produced from a short-slit die [24,28]. The control extrudate had bigger air cells than the extrudate added with 15% oyster mushroom (Figure 4a: iv, and Figure 4b: iv). Besides, Figure 4a: iii, iv and Figure 4b: iii, iv present the presence of a fibrous structure in the extrudates. The 15% oyster mushroom added-extrudates exhibited a defined fibrous structure as compared with the control extrudates. As discussed in the TI section, increasing the oyster mushroom content to 15% increased the TI of the extrudates, which was supported by the SEM images in Figure 4b: iii, iv. Samard and coworkers (2019) reported differences in the formation of air cells and fibrous structure mechanisms based on the type of extruder die used [24]. The low-moisture extrudates produced from a short-slit die had an expanded and porous structure, while the high-moisture extrudates exited from a long cooling die created a dense, layered, and fibrous structure. A previous study conducted by Thadavathi and coworkers (2019) failed to observe the formation of a continuous protein fiber network [5]. This failure might be due to an inadequate protein content to develop protein networks through a single-screw extrusion process. The formation of a protein network in a meat analog involved the stages of unraveled protein native state (interruption of hydrogen bond and van der Waals’ forces) and realignment of linear protein subunits (new crosslinking amide bonds between free-carboxyl and amino residues) within the shear environment of the screw and texturization at the die [34,35,36]. 

## 4. Conclusions

A factorial experiment design was successfully used to determine the suitable barrel temperature of 140 °C, screw speed ranging from 110 to 160 rpm, and oyster mushroom addition at 0%, 7.5%, and 15% for the extrusion of unpuffed extrudates with fibrous structure. Addition of oyster mushroom to the soy protein-based meat analog resulted in a significantly reduced expansion ratio and apparent density but increased the moisture content and WAI. In this study, the apparent density of the extrudates was found to be affected more by the increment of screw speed compared with the oyster mushroom content. The TI of the extrudates increased significantly when the oyster mushroom content increased. However, the screw speed only had a minor effect on the TI of the extrudates. The application of single-screw extrusion equipped with a slit die produced expanded protein extrudates with non-uniform air cell distribution. At the addition of 15% oyster mushroom, the extrudate had a smaller air cell and a defined fibrous structure compared with the control extrudate. Overall, the study could be useful for researchers looking for a better understanding of extrusion flow performance and extrudate physical changes, as well as food manufacturers that aim for the development of meat analogs with improved aesthetic properties based on plant-based mixtures and processing.

## Figures and Tables

**Table 1:** Ingredient formulations at a different percentage of oyster mushroom content.

	Ingredients	Formulations (wb%)				
		1	2	3	4	5
Base mixture	Soy protein concentrate (SP)	50	50	50	50	50
	Isolated soy protein (ISP)	50	50	50	50	50
Additional ingredients (% by total weight of the base mixture)	Wheat flour	2	2	2	2	2
	Soy lecithin	0.4	0.4	0.4	0.4	0.4
	Sodium metabisulfite	0.18	0.18	0.18	0.18	0.18
	Salt	3	3	3	3	3
	Cooking palm oil	10	10	10	10	10
	Distilled water	70	70	70	70	70
	Oyster mushroom	0	7.5	15	25	35

**Table 2:** Extrudability and extrudates’ satisfactory characteristics at different barrel temperatures, screw speeds, and oyster mushroom contents.

Run	Oyster Mushroom (%)	Extrusion Process Variables		Categorization			Summary
		Barrel Temperature (°C)	Screw Speed (rpm)	Extrudability	Extrudate Characteristic	Illustration	
1	0	130	110	Extrudable	1. Intermittent extrudate flow. 2. Soft and easy to break when cool.	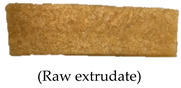	Dissatisfied
2			120				
3			130				
4			140				
5			150				
6			160				
7		140	110	Extrudable	1. Continuous extrudate flow 2. Smooth extrudate surface 3. The fibrous structure is visible	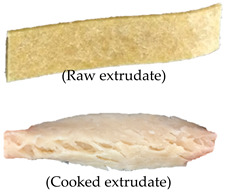	Satisfied
8			120				
9			130				
10			140				
11			150				
12			160				
13		150	110	Extrudable	1. Continuous extrudate flow 2. Extrudate puffed at die	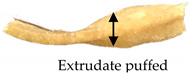	Dissatisfied
14			120				
15			130		3. Intermittent extrudate flow and extrudate shoot out form die	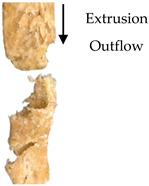	Dissatisfied
16		160	110	Not extrudable	1. Extrudate hardened at die 2. No extrudate collected	Not available	Dissatisfied
17	7.5	140	110	Extrudable	1. Continuous extrudate flow 2. Smooth extrudate surface 3. The fibrous structure is visible	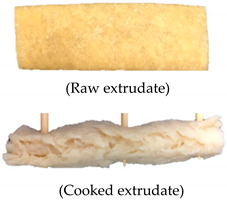	Satisfied
18			120				
19			130				
20			140				
21			150				
22			160				
23	15	140	110	Extrudable	1. Continuous extrudate flow 2. Smooth extrudate surface 3. The fibrous structure is visible	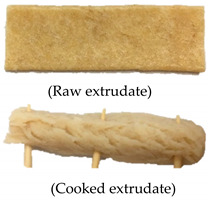	Satisfied
24			120				
25			130				
26			140				
27			150				
28			160				
29	25	140	110	Extrudable	1. Intermittent extrudate flow 2. Extrudate shoot out from die	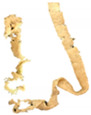	Dissatisfied
30			120				
31			130				
32	35	140	110	Extrudable	1. Intermittent extrudate flow 2. Extrudate shoot out from die	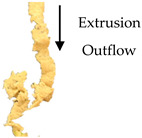	Dissatisfied

**Table 3:** Physical and textural properties of the extrudates.

Run	Barrel Temperature (°C)	Oyster Mushroom Addition (%)	Screw Speed (rpm)	Physical and Textural Properties of the Extrudates				
				ER	$\rho_{\mathrm{app}}\;(\mathrm{kg}/m^{3})$	Moisture Content (%)	WAI (g/g)	TI
1	140	0	110	1.17 ± 0.01	1380.96 ± 10.12	31.40 ± 1.03	2.91 ± 0.10	0.77 ± 0.01
2			120	1.17 ± 0.01	1381.55 ± 19.29	32.33 ± 0.28	3.17 ± 0.08	0.83 ± 0.08
3			130	1.09 ± 0.01	1393.70 ± 6.30	29.49 ± 0.13	3.22 ± 0.07	0.85 ± 0.02
4			140	1.15 ± 0.02	1284.80 ± 8.41	28.79 ± 0.37	3.48 ± 0.01	1.01 ± 0.01
5			150	1.16 ± 0.05	1261.27 ± 13.41	26.99 ± 0.49	3.41 ± 0.06	0.87 ± 0.07
6			160	1.26 ± 0.01	1182.72 ± 6.12	29.56 ± 0.33	3.54 ± 0.03	0.83 ± 0.12
7	140	7.5	110	1.01 ± 0.02	1339.92 ± 6.55	35.64 ± 0.23	3.61 ± 0.08	1.09 ± 0.01
8			120	1.02 ± 0.01	1331.66 ± 2.97	35.71 ± 0.18	3.48 ± 0.12	1.01 ± 0.01
9			130	1.09 ± 0.01	1370.92 ± 17.29	35.49 ± 0.08	3.72 ± 0.08	1.02 ± 0.02
10			140	1.12 ± 0.01	1354.46 ± 2.20	35.29 ± 0.37	3.64 ± 0.04	0.99 ± 0.00
11			150	1.02 ± 0.02	1351.17 ± 10.27	35.08 ± 0.04	3.96 ± 0.02	1.05 ± 0.02
12			160	1.08 ± 0.01	1322.25 ± 5.71	34.77 ± 0.08	3.83 ± 0.02	1.12 ± 0.02
13	140	15	110	0.99 ± 0.01	1327.08 ± 3.20	37.32 ± 0.13	3.76 ± 0.18	1.09 ± 0.02
14			120	0.99 ± 0.01	1302.90 ± 8.74	37.72 ± 0.05	3.77 ± 0.09	1.09 ± 0.12
15			130	0.98 ± 0.01	1315.44 ± 3.51	37.74 ± 0.22	3.86 ± 0.04	1.07 ± 0.03
16			140	1.00 ± 0.01	1285.62 ± 8.97	37.50 ± 0.05	4.04 ± 0.07	0.97 ± 0.02
17			150	1.01 ± 0.02	1298.37 ± 9.09	37.93 ± 0.05	3.97 ± 0.07	0.95 ± 0.01
18			160	1.00 ± 0.01	1272.73 ± 2.94	36.17 ± 0.01	3.92 ± 0.18	1.19 ± 0.04

**Table 4:** Coefficients of factorial regression for the physical properties of the extrudate.

		ER	$\rho_{\mathrm{app}}$	Moisture Content	WAI	TI
*p*-value	OM	0.0001 ****	0.017 *	0.0001 ****	0.0001 ****	0.0001 ****
	SS	0.015 *	0.0001 ****	0.015 *	0.0001 ****	0.059
*R* ^2^		0.85	0.58	0.88	0.94	0.71

*, **** = significant at *p* ≤ 0.05, *p* ≤ 0.0001 respectively. Notes: OM = oyster mushroom addition (%); SS = screw speed (rpm).

**Table 5:** Correlation coefficients among the expansion ratio, apparent density, water absorption index, moisture content, and texturization index.

	ER	$\rho_{\mathrm{app}}$	Moisture Content	WAI	TI
ER	1				
$\rho_{\mathrm{app}}$	−0.175	1			
Moisture Content	−0.820 ****	0.140	1		
WAI	−0.642 ****	−0.349 **	0.678 ****	1	
TI	−0.688 ****	−0.080	0.620 ****	0.657 ****	1

**, **** = significant at *p* ≤ 0.01, *p* ≤ 0.0001, respectively.

## References

[1] Askew K. Meat Analogues: A “Big Opportunity” for Improved Quality. https://www.foodnavigator.com/Article/2019/06/19/Meat-analogues-A-big-opportunity-for-improved-quality.

[2] Asgar M.A., Fazilah A., Huda N., Bhat R., Karim A.A. (2010). Nonmeat protein alternatives as meat extenders and meat analogs. Compr. Rev. Food Sci. Food Saf..

[3] Maida J. Global Meat Substitutes Market 2017–2021. https://www.technavio.com/report/global-meat-substitutes-market.

[4] Rehrah D., Ahmedna M., Goktepe I., Yu J. (2009). Extrusion parameters and consumer acceptability of a peanut-based meat analogue. Int. J. Food Sci. Technol..

[5] Thadavathi Y.L.N., Wassén S., Kádár R. (2019). In-line rheological and microstroctural characterization of high moisture content protein vegetable mixtures in single screw extrusion. J. Food Eng..

[6] Caporgno M.P., Böcker L., Müssner C., Stirnemann E., Haberkorn I., Adelmann H., Handschin S., Windhab E.J., Mathys A. (2020). Extruded meat analogues based on yellow, heterotrophically cultivated *Auxenochlorella protothecoides* microalgae. Innov. Food Sci. Emerg. Technol..

[7] Krintiras G.A., Gadea Diaz J.G., van der Goot A.J., Stankiewicz A.I., Stefanidis G.D. (2016). On the use of the Couette Cell technology for large scale production of textured soy-based meat replacers. J. Food Eng..

[8] Nieuwland M., Geerdink P., Brier P., van den Eijnden P., Henket J.T.M.M., Langelaan M.L.P., Stroeks N., van Deventer H.C., Martin A.H. (2014). Reprint of “Food-grade electrospinning of proteins”. Innov. Food Sci. Emerg. Technol..

[9] Milani T.M.G., Menis M.E.C., Jordano A., Boscolo M., Conti-Silva A.C. (2014). Pre-extrusion aromatization of a soy protein isolate using volatile compounds and flavor enhancers: Effects on physical characteristics, volatile retention and sensory characteristics of extrudates. Food Res. Int..

[10] Ma X., Ryu G. (2019). Effects of green tea contents on the quality and antioxidant properties of textured vegetable protein by extrusion-cooking. Food Sci. Biotechnol..

[11] Croan S.C. (2004). Conversion of conifer wastes into edible and medicinal mushrooms. For. Prod. J..

[12] Synytsya A., Míčková K., Jablonský I., Sluková M., Čopíková J. (2009). Mushrooms of genus *Pleurotus* as a source of dietary fibres and glucans for food supplements. Czech J. Food Sci..

[13] Ahmed M., Abdullah N., Nuruddin N.N. (2016). Yield and nutritional composition of oyster mushrooms: An alternative nutritional source for rural people. Sains Malays..

[14] Wan Rosli W.I., Aishah M.A., Nik Fakurudin N.A., Mohsin S.S.J. (2011). Colour, textural properties, cooking characteristics and fibre content of chicken patty added with oyster mushroom (*Pleurotus sajor-caju*). Int. Food Res. J..

[15] Myrdal Miller A., Mills K., Wong T., Drescher G., Lee S.M., Guinard J.X. (2014). Flavor-enhancing properties of mushrooms in meat-based dishes in which sodium has been reduced and meat has been partially substituted with mushrooms. J. Food Sci..

[16] Banerjee D.K., Das A.K., Banerjee R., Pateiro M., Nanda P.K., Gadekar Y.P., Biswas S., McClements D.J., Lorenzo J.M. (2020). Application of enoki mushroom (*Flammulina velutipes*) stem wastes as functional ingredients in goat meat nuggets. Foods.

[17] Ahirwar R., Jayathilakan K., Reddy K.J., Pandey M.C., Batra H.V. (2015). Development of mushroom and wheat gluten based meat analogue by using response surface methodology. Int. J. Adv. Res..

[18] (FAMA) Federal Agricultural Marketing Authority Kualiti Cendawan Tiram Kelabu Berpandukan, Malaysian Standard (MS 2515:2012). http://www.fama.gov.my/documents/20143/0/cendawan+red.pdf/63676187-392a-48ce-4405-6d4090cb5568.

[19] AOAC (1995). Official Methods of Analysis.

[20] Anderson R.A. (1982). Water absorption and solubility and amylograph characteristics of roll-cooked small grain products. Cereal Chem..

[21] Omohimi C.I., Sobukola O.P., Sarafadeen K.O., Sanni L.O. (2014). Effect of thermo-extrusion process parameters on selected quality attributes of meat analogue from mucuna bean seed flour. Niger. Food J..

[22] Wu M., Sun Y., Bi C., Ji F., Li B., Xing J. (2018). Effects of extrusion conditions on the physicochemical properties of soy protein/gluten composite. Int. J. Agric. Biol. Eng..

[23] Chen F.L., Wei Y.M., Zhang B., Ojokoh A.O. (2010). System parameters and product properties response of soybean protein extruded at wide moisture range. J. Food Eng..

[24] Samard S., Gu B.Y., Ryu G.H. (2019). Effects of extrusion types, screw speed and addition of wheat gluten on physicochemical characteristics and cooking stability of meat analogues. J. Sci. Food Agric..

[25] Bhattacharya S. (1997). Twin-screw extrusion of rice-green gram blend: Extrusion and extrudate characteristics. J. Food Eng..

[26] Arhaliass A., Bouvier J.M., Legrand J. (2003). Melt growth and shrinkage at the exit of the die in the extrusion-cooking process. J. Food Eng..

[27] Lin S., Huff H.E., Hsieh F. (2002). Extrusion process parameters, sensory characteristics, and structural properties of a high moisture soy protein meat analog. J. Food Sci..

[28] Samard S., Ryu G.H. (2019). A comparison of physicochemical characteristics, texture, and structure of meat analogue and meats. J. Sci. Food Agric..

[29] Ning L., Villota R. (1994). Influence of 7s and 11s globulins on the extrusion performance of soy protein concentrates. J. Food Process. Preserv..

[30] Chiang J.H., Loveday S.M., Hardacre A.K., Parker M.E. (2019). Effects of soy protein to wheat gluten ratio on the physicochemical properties of extruded meat analogues. Food Struct..

[31] Fang Y., Zhang B., Wei Y. (2014). Effects of the specific mechanical energy on the physicochemical properties of texturized soy protein during high-moisture extrusion cooking. J. Food Eng..

[32] Zhang W., Li S., Zhang B., Drago S.R., Zhang J. (2016). Relationships between the gelatinization of starches and the textural properties of extruded texturized soybean protein-starch systems. J. Food Eng..

[33] Takei R., Hayashi M., Umene S., Kobayashi Y., Masunaga H. (2016). Texture and microstructure of enzyme-treated chicken breast meat for people with difficulties in mastication. J. Texture Stud..

[34] Huang F.F., Rha C. (1974). Protein structures and protein fibers: A review. Polym. Eng. Sci..

[35] Stanley D.W., deMan J.M. (1978). Structural and mechanical properties of textured proteins. J. Texture Stud..

[36] Arêas J.A.G. (1992). Extrusion of food proteins. Crit. Rev. Food Sci. Nutr..

